# Mechanistic Insights Into How Acupuncture Modulates Host Immunity via the Gut Microbiota‐Macrophage Axis

**DOI:** 10.1002/iid3.70567

**Published:** 2026-11-05

**Authors:** Tao Yang, Mei Li, Xiaowen Wu

**Affiliations:** ^1^ Department of Laboratory Medicine Jiangsu Road Sub‐district Community Health Service Center Qingdao City Shandong Province China; ^2^ Department of Traditional Chinese Medicine Badaxia Sub‐district Community Health Service Center Qingdao City Shandong Province China; ^3^ Department of General Internal Medicine The Integrated Traditional Chinese and Western Medicine Hospital of Shinan District (The People's Hospital of Shinan District) Qingdao City Shandong Province China

**Keywords:** acupuncture, gut microbiota, intestinal barrier, macrophage polarization, neuro‐immune axis, short‐chain fatty acids (SCFAs)

## Abstract

**Background:**

Acupuncture is proposed to operate within the neuro‐endocrine‐immune network. Its therapeutic efficacy is proposed to be partly mediated through the gut microbiota‐macrophage axis. This proposal is based primarily on preclinical findings. This axis may act as a biological transducer that helps sustain systemic homeostasis^1^. This review synthesizes current evidence on how acupuncture may orchestrate a top‐down regulation of the intestinal microenvironment through somato‐vagal circuits, particularly those involving PROKR2‐expressing sensory fibers.

**Materials and Methods:**

This narrative review examined recent preclinical and clinical literature on acupuncture, gut microbiota, metabolites, and macrophage polarization. Studies were selected by relevance and expert judgment. Evidence was organized around the proposed gut microbiota‐macrophage axis.

**Results:**

In preclinical rodent models and some human studies, acupuncture has been reported to suppress pathogens such as *Proteobacteria*. It may also promote symbionts like *Akkermansia muciniphila* and butyrate‐producing taxa. Butyrate promotes anti‐inflammatory M2 macrophage polarization via histone deacetylase (HDAC) inhibition and G‐protein‐coupled receptor signaling. In rodent models, acupuncture has been shown to strengthen barrier integrity. It may upregulate tight junction proteins. It may also prevent lipopolysaccharide (LPS) translocation and suppress M1‐driven cascades. These effects extend distally via the gut‐lung and gut‐brain axes.

**Discussion:**

It is important to note that this proposed cascade is primarily derived from preclinical rodent models, and direct evidence in humans that confirms all steps from vagal activation to macrophage reprogramming remains incomplete. Ultimately, acupuncture may serve as a potential eco‐immunological therapy that restores balance between the microbiome and innate immunity. This framework provides a preliminary conceptual basis for the eventual development of personalized, biomarker‐driven protocols for chronic inflammatory diseases, although this hypothesis requires rigorous causal validation.

## Graphical abstract

Electroacupuncture at specific acupoints (e.g., ST36, ST25) activates somato‑vagal neural circuits via PROKR2‑expressing sensory fibers. This top‑down signal restructures the gut microenvironment by enriching beneficial symbionts (e.g., Akkermansia muciniphila) and strengthening the intestinal barrier. The consequent rise in microbial‑derived butyrate reprograms macrophages toward an anti‑inflammatory M2 phenotype via histone deacetylase inhibition and G‑protein‑coupled receptor signaling. In parallel, the reinforced barrier attenuates lipopolysaccharide translocation, suppressing TLR4/NF‑κB‑mediated M1 macrophage activation. These coordinated actions re‑establish systemic immune homeostasis, exerting therapeutic effects via the gut–brain and gut–lung axes.

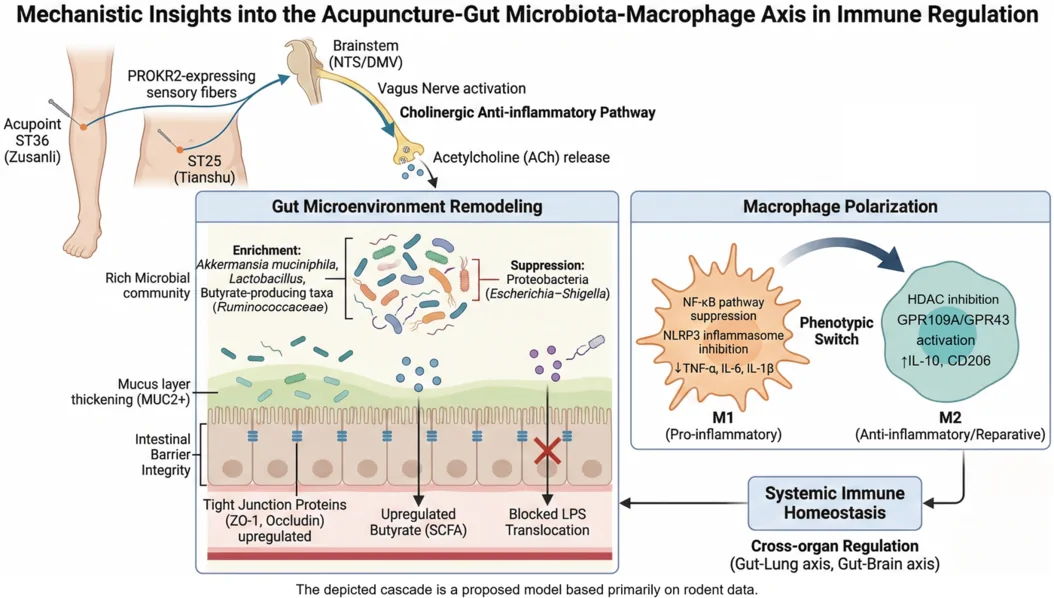

## 1 Introduction

Before delving into the immunomodulatory mechanisms of acupuncture, it is essential to clarify the nomenclature and procedural context of this ancient yet modernizing practice. Acupuncture, a cornerstone of traditional Chinese medicine, involves the insertion of fine, sterile needles into specific anatomical loci known as acupoints (e.g., ST36 Zusanli). Contemporary clinical and experimental protocols frequently employ electroacupuncture (EA), a modified technique wherein a mild electrical current is delivered via the inserted needles to provide quantifiable and sustained stimulation of underlying neural pathways. This distinction is critical, as the frequency and intensity of EA parameters profoundly influence neuro‐immune outcomes. In rodent models, these interventions have been shown to initiate somato‐autonomic reflexes. In rodent models, a specific subset of sensory fibers marked by PROKR2‐Cre expression has been shown to transduce peripheral stimulation into central nervous system signals that regulate distal organ function [1]. However, direct evidence for such fiber‐specific circuits in humans remains lacking, and detailed nerve tracing studies are still incomplete [2]. Establishing this procedural and terminological foundation is crucial. It helps us appreciate how acupuncture may operate as a top‐down modulator within the neuro‐endocrine‐immune network. It may also influence the gut microenvironment.

Acupuncture has evolved from an empirical practice into a scientifically informed intervention that may operate within the neuro‐endocrine‐immune network [1, 3, 4, 5]. This evolution suggests that acupuncture may act as a key modulator within this network. It may act through bidirectional immunomodulation. It may suppress excessive inflammation while preserving innate host defenses [6].

While the neuroanatomical foundations of acupuncture, such as somato‐autonomic neural circuits including the cholinergic anti‐inflammatory pathway [7], have been partially elucidated, the precise peripheral effectors of these signals remain unclear. Although the vagal‐adrenal axis and acetylcholine release are established as critical mediators [8], it is still unknown how these distal signals sustain immunological equilibrium in peripheral tissues. In particular, the intestine functions as the body's largest immune organ and a major microbial reservoir. It may represent a key distal target of acupuncture [9]. Its role in chronic immune regulation remains to be fully elucidated.

The intestinal microenvironment constitutes a critical interface where the host immune system engages with a complex microbial ecosystem. Central to this crosstalk is the gut microbiota‐macrophage axis, a sophisticated biological transducer that converts microbial signals into coordinated immune responses [10]. Within this axis, intestinal macrophages serve as frontline sentinels, exhibiting remarkable functional plasticity [11]. These cells undergo a dynamic training process directed by microbial metabolites. These metabolites act as decisive ligands that instruct macrophage polarization toward either a pro‐inflammatory (M1) or an anti‐inflammatory, reparative (M2) phenotype [12, 13]. Beneficial microbial metabolites, such as butyrate and indole derivatives, promote a tolerogenic and reparative immune tone through epigenetic and receptor‐mediated pathways [14], thereby underpinning systemic immune homeostasis.

This review comprehensively synthesizes recent advances in understanding how acupuncture modulates host immunity through the gut microbiota‐macrophage axis. This is a narrative review; as such, literature selection was based on relevance and expert judgment rather than a predefined systematic search protocol. Specifically, acupuncture may intervene at the level of the gut ecological niche. It may not target isolated immune components alone. In rodent models, this intervention has been reported to remodel the microbial community and its metabolic output [15]. This may direct macrophage polarization toward a homeostatic phenotype [16]. We examine the dual strategy through which acupuncture promotes anti‐inflammatory M2 programs via metabolite upregulation [13] while suppressing pro‐inflammatory M1 drivers through intestinal barrier restoration [17]. Ultimately, this synthesis suggests that acupuncture may function as a distinctive eco‐immunological therapy that harmonizes host immunity by recalibrating foundational microbial‐immune networks, a hypothesis that warrants further causal validation [18].

## 2 Acupuncture May Modulate the Gut Microbiota

Within the framework of the neuro‐endocrine‐immune network, converging evidence suggests that acupuncture may function as a potent biological intervention capable of promoting systemic homeostasis through the remodeling of the gut microecosystem [19, 20, 21]. As the largest immune organ and a major reservoir of microorganisms, the intestine represents a critical distal target for the therapeutic actions of acupuncture. In rodent models, EA has demonstrated the ability to correct microbial dysbiosis in various pathological conditions. This may form a prerequisite for subsequent immune modulation [22].

### 2.1 Dysbiosis Correction by Acupuncture in Multi‐Disease Models

Gut dysbiosis, characterized by reduced microbial diversity, loss of commensal symbionts, and expansion of opportunistic pathogens, is a common feature of numerous systemic diseases. In both preclinical rodent models and clinical studies, acupuncture has been reported to restore key alpha diversity indices, such as the Shannon and Chao1 indices. It may also rebalance microbial composition under multiple pathological conditions [23, 24, 25]. In rodent models of inflammatory bowel disease and irritable bowel syndrome, inflammation‐induced oxidative stress profoundly disrupts the gut flora. In these models, acupuncture was applied at specific acupoints. These included Zusanli (ST36; located on the stomach meridian below the knee), Tianshu (ST25), and Dachangshu (BL25). Acupuncture has been reported to significantly enhance microbial richness and evenness [26]. In obese mice with experimentally induced colitis, electroacupuncture has been shown to suppress intestinal ferroptosis (an iron‐dependent form of programmed cell death) and inflammation. This may occur by activating the Nrf2/HO‐1 signaling pathway. This may establish a physiological niche more favorable for beneficial anaerobic bacteria [27]. In rodent IBS models, acupuncture's mechanisms may extend beyond microbiota modulation. They may also affect neuro‐immune and barrier repair pathways. For instance, in a rat model of diarrhea‐predominant IBS, EA at ST36, ST25, and LR3 has been shown to restore intestinal barrier integrity primarily by inhibiting mast cell activation and downregulating exosomal miRNAs (e.g., miR‐149‐5p), thereby upregulating tight junction proteins [28]. In contrast, other approaches, such as EA at the Baliao points (BL31–BL34), have been reported to alleviate symptoms. This may occur through modulating the gut microbiota‐mast cell‐TRPV1 axis [27].

In rodent models of obesity and metabolic dysregulation, EA at ST36 and Quchi (LI11) has been reported to remodel the gut microbiota. In preclinical studies using diet‐induced obese rats, a notable outcome is the reduction of the Firmicutes‐to‐Bacteroidetes ratio in obese rats [29]. Importantly, a randomized controlled trial involving patients with abdominal obesity supports these findings. It reported that an 8‐week EA regimen significantly reduced waist circumference. It also selectively enriched levels of *Akkermansia muciniphila* [30]. These microbial changes correlate with improved serum lipid profiles and activation of host PPAR signaling pathways [23].

In rodent models, the therapeutic influence of acupuncture may extend to pulmonary immunity via the gut‐lung axis. In ovalbumin‐induced allergic asthma models in mice, acupuncture at points such as Dazhui (GV14), Fengmen (BL12), and Feishu (BL13) has been reported to shift the pulmonary immune response. It may shift from a Th2/Th17‐dominant inflammatory state toward a Th1/Treg‐dominant homeostatic state. This immunologic transition is accompanied by the recovery of *butyrate‐producing bacteria* in the gut [31]. The consequent increase in systemic short‐chain fatty acids, particularly butyrate, may help inhibit NLRP3 inflammasome activation in the lungs. This may reduce airway hyperresponsiveness [31].

Across both preclinical rodent studies and human clinical investigations, a frequently reported taxonomic shift is observed following acupuncture treatment, marked by enrichment of probiotic taxa and suppression of pathogenic ones [32], although it should be noted that this apparent consistency may be influenced by the underreporting of negative or inconclusive findings as discussed in Section 5.2. In rodent models and some human studies, beneficial genera such as Lactobacillus, including species such as L. reuteri and L. johnsonii, have been reported to be frequently enhanced. In mouse models of constipation and insomnia, this increase may facilitate tryptophan metabolism. It may improve gut motility and neurobehavioral outcomes via the aryl hydrocarbon receptor [33]. Similarly, in rat models, acupuncture has been reported to restore *Bifidobacterium* levels. This may strengthen the intestinal barrier and downregulate systemic pro‐inflammatory cytokines [34]. In rodent models and some clinical studies, enrichment of *A. muciniphila* following EA at ST36 has been reported. It has been associated with improvements in stroke recovery, metabolic health, and cognitive function [35, 36]. Conversely, in rodent models, acupuncture has often been reported to reduce members of the phylum Proteobacteria. This includes lipopolysaccharide‐producing genera such as *Escherichia‐Shigella*. In rodent models of acute ischemic stroke, this reduction following acupuncture has been reported to correlate with protection of the blood‐brain barrier and attenuated neuroinflammation [35].

### 2.2 Mechanisms of Modulation From Neural Reflex to Barrier Restoration

Acupuncture may regulate distal gut microbiota through two interconnected mechanisms. These are neural reflex‐driven modifications of the intestinal habitat and direct restoration of intestinal barrier integrity. Together, these pathways may reshape the microenvironment to favor microbial colonization.

The distal therapeutic effect of acupuncture may be largely mediated by specific somato‐autonomic neural circuits. This has been demonstrated primarily in rodent models using optogenetic and electrophysiological approaches. For example, in rodent studies, stimulation at acupoints like ST36 has been shown to activate afferent signals. ST36 is densely innervated by sensory fibers, including those expressing PROKR2‐Cre [1]. Whether similar fiber distributions exist at human acupoints remains to be established. These signals project to key brainstem nuclei, such as the nucleus tractus solitarius and the dorsal motor nucleus of the vagus nerve, ultimately activating vagal efferent pathways. Subsequent acetylcholine release in the gastrointestinal tract may coordinate multiple beneficial effects. It may promote normal gastrointestinal motility and secretion. This may help prevent bacterial overgrowth and maintain physiological stratification of microbial communities along the gut [37]. Moreover, this vagally derived acetylcholine may engage the cholinergic anti‐inflammatory pathway. It may bind to the α7 nicotinic acetylcholine receptor on resident intestinal macrophages. It may suppress the release of pro‐inflammatory cytokines. This action may create a low‐inflammatory microenvironment in the gut lumen. This may be crucial for protecting oxygen‐sensitive beneficial anaerobic bacteria from oxidative stress [38].

In rodent models, acupuncture has been shown to enhance intestinal barrier function. This may modify the host landscape in which microbes reside. In rodent models of metabolic dysfunction and colitis, electroacupuncture has been shown to upregulate expression of key tight junction proteins, including ZO‐1, occludin, and claudin‐1, thereby repairing the paracellular seal between epithelial cells. This repair process may be facilitated through vagus‐dependent mechanisms. For example, vagal activation can promote epithelial α1,2‐fucosylation via fucosyltransferase 2 [39]. This biochemical modification may strengthen the mechanical barrier. It may also provide specific carbohydrate ligands for commensal bacterial adhesion. In rodent models, acupuncture has been reported to reinforce the chemical and physical barrier. It may increase the density of MUC2‐positive goblet cells and stimulate mucus secretion. A robust and well‐structured mucus layer acts as a critical physical shield against pathogen infiltration and serves as a selective nutrient source for mucin‐utilizing commensals such as *A. muciniphila*, promoting their preferential enrichment. An additional regulatory layer involving host microRNAs exists [40]. In rat models, electroacupuncture at acupoints ST36, ST25, and LR3 can modulate the cargo of mast cell‐derived exosomes, regulating levels of specific miRNAs such as miR‐149‐5p and miR‐22‐5p. These miRNAs help maintain barrier integrity by preventing degradation of tight junction proteins, thereby preserving the luminal physicochemical environment [28].

In summary, acupuncture has been shown to orchestrate a coordinated response in rodent models. This response originates from somatosensory stimulation. It integrates within the brainstem. It executes via the vagus nerve to modulate gut physiology and inflammation [41]. In rodent models, it may directly strengthen the intestinal wall. These dual actions may collectively modify niche conditions. They may render the gut environment more conducive to a symbiotic microbiota and less favorable for pathobionts. This may underpin the observed microbial remodeling [26].

## 3 The Critical Link Between Microbiota Metabolites and Macrophages

The translation of gut microbiota compositional shifts into systemic immune modulation is governed by a molecular dialogue between microbial metabolites and host immune sentinels, particularly macrophages. These bacterial by‐products act as decisive ligands that direct macrophage polarization toward either a pro‐inflammatory (M1) or an anti‐inflammatory, regulatory (M2) phenotype [12]. Although the M1/M2 dichotomy is a useful heuristic for organizing this review, macrophage functional states *in vivo* actually exist along a continuous spectrum, as discussed in Section 5.2.

Short‐chain fatty acids, especially butyrate, are key metabolites from dietary fiber fermentation. Evidence from both *in vitro* macrophage cultures and *in vivo* animal models demonstrates that butyrate promotes M2 polarization through two complementary mechanisms. First, it acts as an epigenetic modulator by inhibiting class I and II histone deacetylases (HDACs) [42]. This enhances histone acetylation, which promotes anti‐inflammatory gene transcription and reduces macrophage responsiveness to inflammatory triggers. In parallel, butyrate engages specific G‐protein‐coupled receptors. Its binding to GPR109A suppresses the LPS‐induced NLRP3 inflammasome activation and pyroptosis, while signaling through GPR41 and GPR43 inhibits the NF‐κB pathway [43]. This receptor‐mediated signaling dampens the production of cytokines such as IL‐6 and IL‐12, while upregulating M2 markers like CD206 and stimulating IL‐10 secretion. Figure 1 illustrates the dual signaling mechanisms by which butyrate promotes M2 polarization through HDAC inhibition and GPR109A activation.

**Figure 1:**
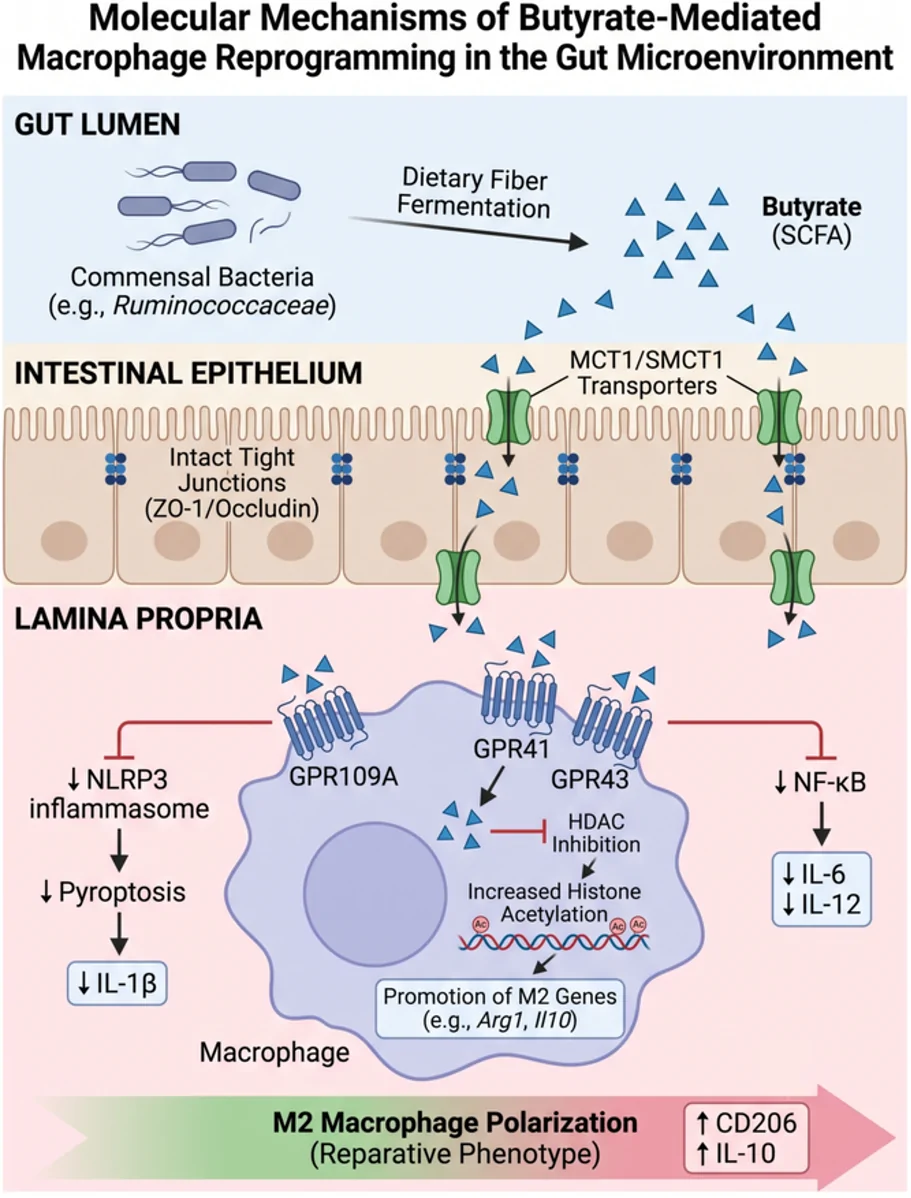
Molecular mechanisms of butyrate‐mediated macrophage reprogramming in the gut microenvironment. Commensal‐derived butyrate crosses the epithelial barrier via MCT1/SMCT1 transporters to target lamina propria macrophages. It promotes M2 polarization through two main signaling routes. First, butyrate acts as an HDAC inhibitor. This increases histone acetylation, upregulates reparative genes such as Arg1 and Il10, and supports an anti‐inflammatory phenotype. Second, butyrate activates G‐protein‐coupled receptors. GPR109A signaling suppresses LPS‐induced NLRP3 inflammasome activation and pyroptosis. GPR41 and GPR43 signaling inhibit the NF‐κB pathway. Together, these pathways reduce IL‐6 and IL‐12, increase CD206, and stimulate IL‐10 secretion. The cAMP step has been removed to match the prose.

In contrast, a state of dysbiosis and barrier breach leads to the translocation of pro‐inflammatory microbial components, most notably lipopolysaccharide (LPS). This condition, termed metabolic endotoxemia, is a primary driver of M1 polarization [44, 45]. LPS acts as a classic pathogen‐associated molecular pattern by binding to Toll‐like receptor 4 (TLR4) on macrophages. This interaction triggers the MyD88‐dependent signaling cascade, resulting in the nuclear translocation of NF‐κB and the subsequent robust production of pro‐inflammatory mediators, including TNF‐α, IL‐1β, IL‐6, and IL‐23 [45]. The sustained activation of this TLR4/NF‐κB axis and the resultant M1 phenotype are central to the pathogenesis of chronic inflammatory diseases [45].

Beyond these primary actors, other microbiota‐derived secondary metabolites provide additional layers of immunoregulation. Tryptophan metabolites, such as indole derivatives produced by species like *Lactobacillus*, serve as ligands for the aryl hydrocarbon receptor (AhR) [46]. AhR activation initiates the AhR/CYP1A1 signaling axis within macrophages [47], promoting an M2‐like functional state, attenuating T helper cell‐driven inflammation, and fostering regulatory T cell expansion [46, 48]. Similarly, gut microbiota‐transformed secondary bile acids, including deoxycholic acid and lithocholic acid, modulate inflammation through receptors like TGR5 and the nuclear Farnesoid X Receptor (FXR). TGR5 activation induces cAMP‐PKA signaling that dismantles the NLRP3 inflammasome [42], while FXR activation antagonizes the NF‐κB pathway, collectively suppressing inflammatory cytokine release and tissue damage [49].

Thus, the gut microbiota exerts precise control over systemic immunity through a spectrum of metabolites. Beneficial metabolites like butyrate and indoles enforce a tolerant, reparative immune tone via epigenetic and receptor‐mediated pathways [50]. Conversely, pathological components like LPS ignite potent inflammatory responses. This dynamic metabolite‐macrophage axis constitutes the essential biological link through which the ecological state of the gut microbiota directly dictates the landscape of host immune homeostasis.

## 4 Potential Modulation of Host Immunity by Acupuncture via the Gut Microbiota‐Macrophage Axis

Acupuncture is proposed to regulate systemic immune homeostasis through its role as a potential master regulator of the gut microbiota‐macrophage axis. This involves two coordinated strategies: promoting anti‐inflammatory pathways while suppressing pro‐inflammatory triggers.

### 4.1 Proposed Promotion of M2 Polarization via SCFA Upregulation

Acupuncture is proposed to initiate a top‐down, neuro‐reflexive cascade. This cascade may culminate in the anti‐inflammatory reprogramming of macrophages. This process is thought to be primarily mediated by the microbial metabolite butyrate. As established in rodent models, stimulation at key acupoints such as ST36 (Zusanli) has been shown to activate specific sensory neurons. These include PROKR2‐Cre fibers. They relay signals to the vagal nuclei in the brainstem [1]. While these findings provide a plausible neuroanatomical basis, direct confirmation in humans is currently lacking, and the translational relevance of PROKR2‐expressing circuits requires further investigation. The subsequent increase in vagal efferent output may optimize the gastrointestinal milieu. It may regulate motility. Through the cholinergic anti‐inflammatory pathway, it may establish a low‐inflammatory, anaerobic niche. This vagally modulated habitat may foster the selective expansion of commensal, butyrate‐producing taxa such as Ruminococcaceae and *Roseburia*. This may lead to a significant rise in colonic and systemic levels of short‐chain fatty acids (SCFAs). Butyrate may be particularly elevated [29]. Supporting human evidence comes from a randomized controlled trial in patients with functional constipation. This trial reported that acupuncture significantly increased fecal butyrate concentrations alongside clinical improvement [33].

The increased butyrate may then function as a potent signaling molecule to reprogram macrophages [43, 51]. It may converge to suppress NLRP3 inflammasome and NF‐kB signaling. This is thought to drive an M2 phenotype. This shift is suggested to contribute to the systemic dampening of inflammation. However, direct evidence in humans that links this cascade to clinical benefit remains incomplete.

### 4.2 Proposed Inhibition of M1 Polarization via Gut Barrier Repair

Simultaneously, acupuncture may counteract the key drivers of pro‐inflammatory macrophage polarization. It may contribute to the restoration of intestinal barrier integrity. This may prevent the leakage of inflammatory stimuli into systemic circulation. This mechanism primarily addresses the state of metabolic endotoxemia, a condition prevalent in chronic diseases like obesity, where increased gut permeability allows luminal lipopolysaccharide (LPS) from pathobionts such as Proteobacteria to translocate into the bloodstream [44].

In an HFD‐induced obese mouse model of colitis/IBD, electroacupuncture at BL25 and ST25 has been shown to strengthen the intestinal barrier. It upregulated the expression of tight junction proteins, including ZO‐1 and Occludin, and ameliorated the loss of goblet cells [27]. This repair process may be mediated, in part, through vagus nerve‐dependent upregulation of fucosyltransferase 2 (Fut2). This may enhance mucosal defense. By potentially restoring intestinal barrier integrity, acupuncture is thought to physically limit the translocation of LPS into the lamina propria and the systemic circulation. Consequently, macrophages may be shielded from the persistent activation triggered by LPS binding to TLR4. This blockade of the downstream TLR4/MyD88/NF‐kB signaling pathway may significantly reduce the induction of the pro‐inflammatory M1 macrophage phenotype. The attenuation of this pathway may lead to a marked decrease in the systemic production and release of potent pro‐inflammatory cytokines, such as TNF‐α and IL‐6. This may contribute to the resolution of chronic inflammation. The relevance of this barrier effect to human chronic inflammatory conditions, however, awaits further investigation.

### 4.3 Local and Systemic Effects: Cross‐Organ Immune Regulation

In rodent models, the immunomodulatory signals generated through the acupuncture‐microbiota‐macrophage axis may extend beyond the intestinal tract. They may exert regulatory effects in remote organs. This may demonstrate the systemic scope of this therapeutic approach.

Within the intestinal lumen, this axis may be crucial for maintaining local homeostasis. It may strike a balance between immune tolerance and defense. In experimental mouse models of ulcerative colitis, acupuncture has been reported to facilitate the resolution of local inflammation. It may help prevent mucosal damage. This may occur by mitigating epithelial oxidative stress and inhibiting pathological processes such as ferroptosis [52].

In rodent models, this gut‐centric modulation may influence distal organs via established physiological axes. In the context of the gut‐lung axis, acupuncture‐induced elevations in gut‐derived SCFAs may enter the systemic circulation. In septic mouse models, these metabolites may inhibit the activation of alveolar macrophages and the NLRP3 inflammasome upon reaching the lungs. This may reduce airway hyperresponsiveness [53]. Similarly, via the gut‐brain axis, acupuncture‐mediated repair of the intestinal barrier may reduce the translocation of pro‐inflammatory mediators into the circulation. This attenuation of peripheral inflammatory load may subsequently decrease the activation of brain‐resident macrophages (microglia). This may lead to diminished neuroinflammation and support neuronal recovery in rodent models of ischemic stroke [24, 35]. The systemic immunomodulatory effects of this axis are summarized in Figure 2. This figure outlines how acupuncture‐mediated gut restoration may attenuate neuroinflammation and airway hyperresponsiveness via the gut‐brain and gut‐lung axes, respectively.

**Figure 2:**
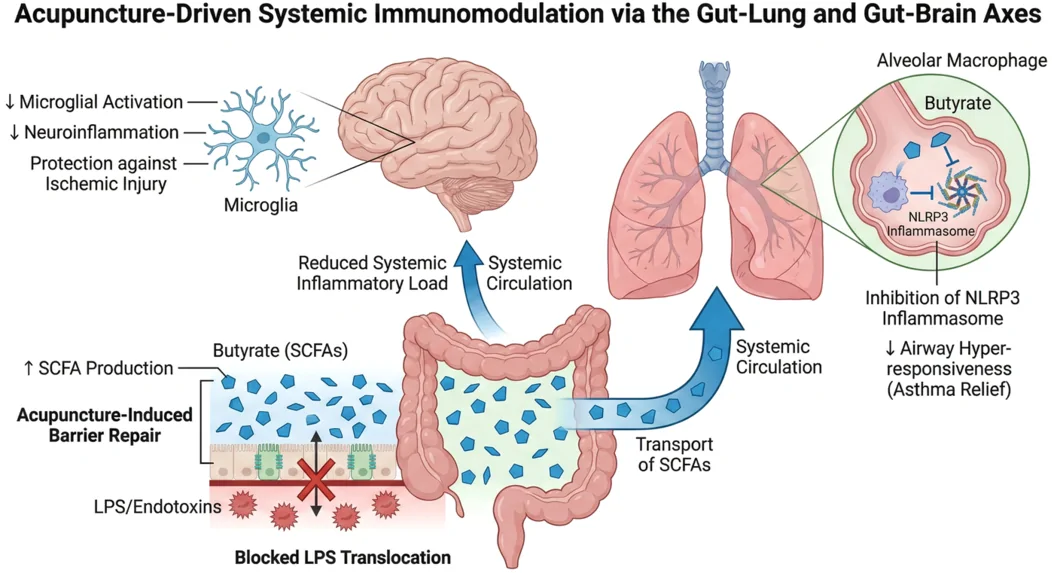
Acupuncture‐driven systemic immunomodulation via the gut‐lung and gut‐brain axes. The depicted cascade is a proposed model based primarily on rodent data. In rodent models, acupuncture has been reported to strengthen the intestinal barrier. It may prevent LPS translocation and boost SCFA production. This may reduce systemic inflammatory load. This may suppress microglial activation and neuroinflammation in the brain. Concurrently, circulating SCFAs may inhibit the NLRP3 inflammasome in pulmonary alveolar macrophages. This may relieve airway hyperresponsiveness in the lungs.

Acupuncture may achieve systemic immunomodulation by targeting the gut ecological niche. It may not target immune components in isolation. This intervention may remodel the microbial community. This may generate a repertoire of metabolites that may direct macrophage polarization. The coordinated upregulation of SCFA‐driven M2 programs and suppression of LPS‐triggered M1 pathways may enable the restoration of immune equilibrium. This may occur locally within the intestinal mucosa and systemically across distant organs. This mechanism suggests that acupuncture may act as a unique eco‐immunomodulator. It may harmonize host immunity through foundational microbial networks.

## 5 Discussion

Skepticism toward acupuncture often focuses on mechanistic plausibility and protocol heterogeneity. This review suggests a balanced view. Current evidence does establish a plausible somato‐vagal circuit and reproducible compositional shifts in the gut microbiota [6, 18]. Skepticism toward acupuncture often focuses on mechanistic plausibility and protocol heterogeneity. This review suggests a balanced view. Current evidence does establish a plausible somato‐vagal circuit and reproducible compositional shifts in the gut microbiota.

As summarized in Table 1, accumulating preclinical and clinical studies across a spectrum of inflammatory and metabolic diseases have reported a consistent paradigm. Acupuncture, particularly at key acupoints such as ST36, may remodel the gut microbial ecosystem and its metabolic output. This remodeling is characterized by the enrichment of beneficial taxa (e.g., *A. muciniphila*, *Lactobacillus*, butyrate producers) and suppression of pathobionts (e.g., Proteobacteria). It may subsequently orchestrate a shift in macrophage polarization from a pro‐inflammatory (M1) to an anti‐inflammatory, reparative (M2) phenotype. This may occur through distinct yet converging molecular pathways. This table provides a synthesized overview of the disease‐specific evidence linking acupuncture‐induced microbial changes to immune reprogramming.

**Table 1:** Summary of preclinical and clinical studies on acupuncture modulating the gut microbiota‐macrophage axis.

Disease/Model	Acupoint (s)	Key gut microbiota changes	Macrophage phenotype shift	Key molecular pathways involved	Species	Study design	Evidence tier	Reference(s)
Ischemic stroke	ST36 (Zusanli)	↑ *Lactobacillus*, ↑ *Bacteroidales*, ↑ *Prevotellaceae*, ↑ *Butyrivibrio*, ↑ SCFA producers (e.g., *Lachnospiraceae*), ↑ anti‐inflammatory genera (e.g., *Eubacterium*); ↓ pathogens (e.g., *Enterococcus faecalis*)	Not directly assessed as M1/M2; reduced neuroinflammation and systemic inflammation	Vagus nerve activation; Fut2‐mediated α1,2‐fucosylation; restored gut barrier (Muc2, ZO‐1, occludin, claudin‐1); reduced neuroinflammation	Rat	Animal model, ischemic stroke (MCAO/reperfusion); vagotomy and FMT validation	Tier 2	[35]
Inflammatory bowel disease (IBD)/Colitis	ST36, ST25 (Tianshu), BL25 (Dachangshu)	↑ Microbial diversity (Shannon/Chao1); ↑ *Lactobacillus* spp.; ↑ *Bifidobacterium*	Not directly assessed; reduced pro‐inflammatory cytokines (refs [27, 52])	Nrf2/HO‐1 activation; ↓ Ferroptosis; ↑ Tight junctions (ZO‐1, Occludin)	Mouse	HFD‐induced obese mice (ref [27]); DSS‐induced colitis mice (ref [52])	Tier 2	[27, 52]
Obesity/Type 2 diabetes	ST36, LI11 (Quchi)	↓ Firmicutes/Bacteroidetes ratio; ↑ *Akkermansia muciniphila*; ↑ SCFA producers	M1 → M2 (in adipose tissue)	PPAR‐γ signaling; ↑ Adipose tissue browning; ↓ Metabolic endotoxemia	Rat and human	Diet‐induced obese rat model; randomized controlled trial (human)	Tier 2/Tier 3	[29, 30, 78]
Irritable bowel syndrome (IBS)	ST36, ST25, LR3	Modulated composition; ↑ Microbial richness	Associated with mast cell stabilization (innate immune regulation)	CRF/CRF‐R1 signaling; mast cell‐derived exosomal miR‐149‐5p; downregulation of tight junction proteins (ZO‐1, Occludin, Claudin‐1)	Rat	IBS‐D rat model	Tier 2	[28]
Functional constipation	ST25 (Tianshu), ST37 (Shangjuxu)	↑ *Lactobacillus reuteri*	Not directly assessed; immune modulation via metabolite signaling	Tryptophan metabolism; indole‐3‐acetamide, indole‐3‐acetic acid, indole‐3‐carboxaldehyde; AhR signaling	Mouse and rat	Tryptophan metabolism; indole‐3‐acetamide, indole‐3‐acetic acid, indole‐3‐carboxaldehyde; AhR signaling	Tier 2	[26, 33]
Insomnia	Back‐Shu points (Xinshu, Ganshu, Pishu, Feishu, Shenshu)	↑ *Lactobacillus johnsonii*, ↑ *Ligilactobacillus murinus*	Not directly assessed; immune modulation via metabolite signaling	Tryptophan and glutamate metabolism; SCFA synthesis; G protein‐coupled receptor signaling	Rat	PCPA‐induced insomnia rat model	Tier 2	[26]
Sepsis/MODS	ST36, RN4 (Guanyuan)	Remodeling of gut microbiota; Reduced dysbiosis	↓ M1 hyperactivation (systemically in lungs, colon, etc.)	Vagal‐adrenal axis; ↓ NLRP3 inflammasome; ↓ MAPK signaling	Mouse and human	Septic mouse model; single retrospective propensity‐matched cohort study	Tier 2/Tier 3	[53, 80]
Metabolic Dysfunction‐Associated Fatty Liver	ST36	Improved dysbiosis; Barrier enhancement	Hepatic macrophage modulation (M1 → M2)	α7nAChR‐mediated HO‐1/p38 MAPK/NF‐κB pathway	Mouse	Mouse model of MAFLD; randomized, single‐blind, controlled animal study	Tier 2	[38]
Diarrhea‐predominant IBS	ST36 (Zusanli), ST25 (Tianshu), LR3 (Taichong)	Modulated microbiota; Barrier restoration	Associated with attenuated innate immune activation	Mast cell exosomal miRNA (miR‐149‐5p) regulation	Rat	IBS‐D rat model (CUMS + Senna)	Tier 2	[28]

*Note:* Arrows: ↑ indicates increase or enrichment; ↓ indicates decrease or suppression; → indicates polarization shift. This table integrates findings from both animal models and human clinical trials referenced in the manuscript. Specific acupoints are listed where explicitly stated in the source. Selection note: The studies summarized in this table are representative examples drawn from the literature cited in this narrative review. They were selected to illustrate the range of disease models and mechanistic pathways discussed in the text and do not represent a systematic extraction of all available studies. Tier definitions follow Section 5.1.1. Tier 1 = observational rodent studies. Tier 2 = mechanistic or causal studies. Tier 3 = clinical studies.

Abbreviations: HO‐1, Heme Oxygenase‐1; LPS, Lipopolysaccharide; MAPK, Mitogen‐Activated Protein Kinase; miRNA, microRNA; MODS, Multiple Organ Dysfunction Syndrome; NF‐κB, Nuclear Factor Kappa B; NLRP3, NOD‐, LRR‐ and Pyrin Domain‐Containing Protein 3; Nrf2, Nuclear Factor Erythroid 2–Related Factor 2; PPAR‐γ, Peroxisome Proliferator‐Activated Receptor Gamma; SCFAs, Short‐Chain Fatty Acids; TLR4, Toll‐like Receptor 4; TRPV1, Transient Receptor Potential Vanilloid 1; α7nAChR, α7 Nicotinic Acetylcholine Receptor.

### 5.1 Critical Appraisal of the Evidence Base

The literature includes a heterogeneous mix of study designs, from associative rodent microbiome profiling to human randomized controlled trials. A rigorous interpretation requires explicit stratification by the strength of causal inference each design permits.

#### 5.1.1 Tiered Evidence: From Hypothesis Generation to Clinical Translation

The evidence can be organized into three tiers. Tier 1 comprises observational studies in rodent models that document correlations between acupuncture, microbial shifts, and immune outcomes [24, 29]. These studies demonstrate association, not causation. For example, the coexistence of increased *Akkermansia* and reduced colitis following electroacupuncture does not prove that microbial remodeling drives the therapeutic effect [35]. Tier 2 consists of mechanistic studies that directly test causality. A limited number have used subdiaphragmatic vagotomy, α7 nicotinic receptor blockade, or antibiotic‐mediated depletion to show that the vagus nerve and an intact microbiome are required for certain acupuncture effects [1, 35]. However, more rigorous models, such as germ‐free animals reconstituted with microbiota from treated donors, remain sparse. Tier 3 includes clinical studies (randomized controlled trials, meta‐analyses) that provide human efficacy data, but mechanistic depth is limited, often relying on correlative analyses of fecal metabolites and circulating cytokines rather than direct causality testing [30, 54]. A detailed mapping of each mechanistic claim to its reference(s), species, study design, and evidence tier is provided in Supplementary Table S1, which follows the tiered framework described above.

#### 5.1.2 Heterogeneity in Electroacupuncture Parameters

A major limitation is the marked heterogeneity in stimulation parameters across studies. Frequency (2–100 Hz), intensity, pulse duration, and session length are inconsistently reported and rarely systematically compared [55, 56]. Low frequency (2 Hz) and high frequency (100 Hz) electroacupuncture activate distinct neuropeptide pathways, but whether these differences translate into divergent effects on the gut ecosystem remains largely unexplored [1]. No study to date has systematically optimized electroacupuncture parameters (e.g., frequency, intensity, pulse duration) for maximal short‐chain fatty acid induction or M2 polarization. Future work must prioritize parametric dose‐response studies to establish standardized, reproducible protocols [57].

#### 5.1.3 Underexplored Validation Models

The causal chain linking acupuncture to immune modulation via the gut microbiota remains incompletely validated. The gold standard approach, fecal microbiota transplantation from treated donors to untreated recipients, has been employed in only a few studies, primarily in stroke and metabolic disease models [35]. Experiments using germ‐free mice or narrow‐spectrum antibiotics to deplete specific microbial clades are notably absent. Without such rigorous designs, the possibility that acupuncture exerts direct neuro‐immune effects independent of the microbiome, or that microbial changes are merely epiphenomena, cannot be excluded [3, 6]. Addressing these gaps through well‐controlled causality studies is essential to elevate the gut microbiota‐macrophage axis from a correlative framework to a validated mechanism [17].

The majority of mechanistic evidence for acupoint‐specific neural circuits (e.g., PROKR2‐Cre fibers at ST36) derives from rodent studies using optogenetic and viral tracing techniques. Human acupoint neuroanatomy may differ substantially, and no study has directly validated the existence or functional role of analogous fiber populations in humans. Thus, claims of acupoint specificity should be interpreted with caution until confirmed in human neuroimaging or nerve block studies [1, 58, 59].

### 5.2 Limitations, Conflicting Evidence, and Alternative Explanations

Most cited studies report positive findings, yet negative or inconclusive results are underpublished [32, 60]. Sham‐controlled trials often show modest specific effects of acupuncture beyond placebo, and a substantial portion of clinical benefit may arise from expectation and stress reduction [61, 62, 63]. Acupuncture has been reported to lower psychological stress and enhance parasympathetic tone. These effects may independently improve gut barrier function and reduce low‐grade inflammation. This may confound attribution to microbiota changes alone. Few studies have rigorously compared verum versus sham acupuncture with microbiome endpoints [64, 65], and alternative pathways (e.g., stress‐induced immune modulation) are rarely controlled for. Acknowledging these limitations and reporting null findings would improve the balance and credibility of the evidence base.

Observed associations between acupuncture, microbiota shifts, and immune outcomes do not establish causality; microbiota changes may be a consequence rather than a driver of reduced inflammation [66, 67]. Moreover, most studies infer SCFA changes from fecal levels without direct tissue measurement [68, 69, 70], and the M1/M2 macrophage polarization paradigm is an oversimplification of a continuous spectrum of functional states [71, 72, 73]. Direct human evidence linking all steps of the proposed axis (vagal activation → specific microbial taxa → elevated SCFAs → macrophage reprogramming) remains fragmentary [32].

### 5.3 Inflammatory Bowel Disease

Inflammatory bowel disease, encompassing ulcerative colitis (UC) and Crohn's disease, is characterized by a vicious cycle of epithelial barrier disruption, microbial translocation, and sustained pro‐inflammatory (M1) macrophage activation [10, 74]. Clinical evidence positions acupuncture, particularly as an adjunctive therapy, to potentially enhance conventional treatments like 5‐aminosalicylic acid. At the clinical level, a large‐scale network meta‐analysis of 76 randomized controlled trials (RCTs) involving 7484 participants reported that acupuncture combination therapy significantly improved clinical Mayo scores. It also reported improved endoscopic mucosal healing [75]. The meta‐analysis included a risk of bias assessment. These findings should be interpreted with caution because the synthesis drew largely on Chinese‐language databases. Publication language, risk of bias, and effect size may influence the estimates, as discussed in Section 5.2 and reference [61]. In clinical studies, specific modalities such as warm acupuncture and auricular acupressure have been reported to reduce systemic levels of pro‐inflammatory cytokines, including IL‐6 and TNF‐α [27].

At the mechanistic level, studies in rodent experimental colitis models have shown that electroacupuncture at acupoints ST36 and ST25 may facilitate a phenotypic switch in intestinal macrophages. This switch may be from a pro‐inflammatory M1 state to a tissue‐reparative M2 phenotype [7]. This reprogramming may be driven by the activation of the Nrf2/HO‐1 signaling pathway [27]. Concurrently, this axis activity may suppress colonic ferroptosis and upregulate the expression of tight junction proteins. This may restore epithelial barrier integrity. Thus, in rodent colitis models, acupuncture may intervene in the pathogenic cycle of IBD. It may restore epithelial barrier integrity and reprogram macrophages toward an M2 reparative phenotype. This may disrupt the self‐perpetuating loop of inflammation driven by M1 activation and microbial translocation.

### 5.4 Metabolic Diseases

Obesity and Type 2 Diabetes Mellitus (T2DM) are systemic inflammatory states where gut dysbiosis contributes to metabolic endotoxemia, driving M1 macrophage infiltration into white adipose tissue (WAT) and the pancreas [76]. Acupuncture may intervene in this cascade by remodeling the inflammatory microenvironment. In obese rat models, EA at Zusanli (ST36) and Quchi (LI11) has been shown to reduce body weight, visceral adiposity, and crucially, the recruitment of pro‐inflammatory macrophages into WAT [77].

The therapeutic benefits may be mediated through a coherent “gut microbiota‐metabolite‐barrier‐inflammation” axis [78]. In HFD‐induced obese rat models, acupuncture has been reported to enrich beneficial genera such as *Akkermansia* and *Bifidobacterium*. This may lead to increased production of SCFAs like acetate and propionate [77]. These microbial metabolites may subsequently activate host PPAR‐γ signaling. This may promote adipose tissue browning, improve insulin sensitivity, and rebalance the M1/M2 macrophage ratio in metabolic tissues [78].

### 5.5 Functional Constipation

Functional constipation (FC) is a prevalent gastrointestinal disorder characterized by disrupted gut motility, often associated with alterations in the gut microbiota and their metabolic output. Acupuncture, particularly electroacupuncture (EA), has been reported to improve symptoms in FC patients and model animals. Emerging research provides a detailed mechanistic link within the gut microbiota‐macrophage axis framework.

A pivotal preclinical study by Wang et al. [33] revealed that EA at acupoints ST25 (Tianshu) and ST37 (Shangjuxu) alleviated loperamide‐induced constipation in mice. This may occur by orchestrating a precise “host miRNA‐bacteria‐metabolite” cascade. The treatment was found to upregulate host‐derived miR‐205‐5p in colonic tissues, which in turn promoted the intestinal expansion of *Lactobacillus reuteri*. This specific microbial shift led to a marked increase in bacterial tryptophan metabolism, elevating the levels of key metabolites including indole‐3‐acetamide, indole‐3‐acetic acid (IAA), and indole‐3‐carboxaldehyde (I3A).

These tryptophan derivatives are established ligands for the AhR. AhR activation in immune cells, including macrophages, is thought to drive an anti‐inflammatory, regulatory phenotype. This is analogous to M2 polarization. It may also modulate neuro‐immune circuits in the gut. Thus, this study delineates a coherent pathway: EA → upregulation of miR‐205‐5p → enrichment of *L. reuteri* → production of AhR‐activating tryptophan metabolites → potential promotion of a tolerogenic immune milieu and improved gut motility. This mechanism suggests that acupuncture may exert systemic effects. It may do so by initiating a host‐derived molecular signal that may reshape a microbial metabolite axis. This may have immunomodulatory consequences.

### 5.6 Sepsis/Acute Injury

In critical conditions like sepsis, uncontrolled innate immune activation leads to a lethal “cytokine storm” and multi‐organ dysfunction syndrome (MODS). Acupuncture emerges as a potential neuromodulatory strategy for systemic immunomodulation. A single retrospective propensity‐matched cohort study reported that early intervention with EA at Zusanli (ST36) and Guanyuan (RN4) was associated with lower 28‐day mortality in sepsis patients with acute gastrointestinal injury [79]. This finding comes from one retrospective study, so it should be interpreted with caution.

In preclinical rodent models of sepsis‐induced MODS, the protective mechanism involves dampening the hyperactivation of macrophages across organs. In rodent models of MODS, acupuncture has been reported to inhibit key inflammatory pathways, including the NLRP3 inflammasome and MAPK signaling, in the lungs and colon [53]. This effect may be largely mediated by stimulating PROKR2‐Cre neurons. These neurons may activate the vagus‐adrenal anti‐inflammatory axis to release dopamine. This may suppress systemic macrophage activation [7]. Furthermore, in rodent models, acupuncture‐induced microbial signals may exert remote tissue protection. They may regulate alveolar macrophages in acute lung injury and microglia in sepsis‐associated encephalopathy. This may occur primarily through inhibition of the TLR4/NF‐κB cascade. By stabilizing the systemic immune response during the hyper‐inflammatory phase, acupuncture may offer a complementary strategy. It may help control the cytokine storm and improve outcomes in acute critical illness [7].

## 6 Future Perspectives and Challenges

### 6.1 Establishing Rigorous Causal Validation

Current evidence primarily demonstrates a correlation rather than causation between acupuncture‐induced microbial shifts and immune outcomes. Future research should prioritize study designs that establish both necessity and sufficiency. For example, fecal microbiota transplantation (FMT) from acupuncture‐treated donors into germ‐free models can help determine whether microbial changes alone are sufficient to transfer therapeutic effects. Equally crucial are direct ablation studies employing macrophage depletion (e.g., clodronate liposomes) and targeted neural interruption (e.g., subdiaphragmatic vagotomy) to confirm the indispensable role of the gut‐immune interface versus secondary neuromodulation.

### 6.2 Decoding Acupoint and Context Specificity

The principle of acupoint specificity implies distinct biological signatures, yet its mapping onto the microbiota‐macrophage axis remains incomplete [80, 81]. Emerging data suggest a functional divergence: distal limb points (e.g., ST36) primarily engage long‐range somato‐vagal reflexes, while abdominal points (e.g., ST25) may exert more direct effects on the local intestinal niche and enteric nervous system [7]. Furthermore, the microbial‐immune response to a given acupoint is context‐dependent, varying with the underlying pathology [81]. Elucidating these differential and conditional effects is fundamental for developing personalized treatment protocols [80].

### 6.3 Advancing Standardization and Biomarker‐Driven Parameterization

Clinical translation is hindered by a lack of quantifiable, optimized stimulation protocols. Significant heterogeneity exists across studies regarding acupoint selection and stimulation parameters [55]. Specific electrical parameters, including frequency, intensity, and waveform, are known to differentially influence neuro‐immune outcomes, with frequency‐specific effects being particularly critical [57]. Future work must systematically quantify how these physical variables shape the production of key immunomodulatory microbial metabolites, such as SCFAs [82]. Concurrently, developing an integrated biomarker system that combines metagenomic, metabolomic, and immunophenotyping data is essential to objectively monitor treatment response and dynamically adjust parameters for optimal efficacy [83].

## 7 Conclusion

Acupuncture has undergone a significant paradigm shift. It is transitioning from a purely empirical modality to a scientifically informed intervention. This intervention may be grounded in the neuro‐endocrine‐immune network [84]. Recent evidence suggests that the therapeutic efficacy of acupuncture may be partly mediated by the gut microbiota‐macrophage axis [20, 53]. This axis may act as a biological transducer. In rodent models, acupuncture has been shown to activate specific somato‐vagal circuits. These circuits involve sensory fibers such as those expressing PROKR2 [7, 35]. Acupuncture may orchestrate a top‐down regulation of the intestinal microenvironment. Whether these circuits are directly translatable to humans remains an open question. This neural drive may optimize the microbial ecosystem. It may suppress opportunistic pathogens such as Proteobacteria. It may also foster a protective niche for symbionts like *A. muciniphila* and various butyrate‐producing taxa [35, 85].

The integration of these microbial shifts into host immunity may occur through a precise molecular dialogue. The upregulation of SCFAs may serve as a signal for macrophage reprogramming [51, 86]. Butyrate may act as a principal actor. This process may promote the anti‐inflammatory M2 phenotype via epigenetic histone deacetylase (HDAC) inhibition and G‐protein‐coupled receptor signaling [87]. Concurrently, the restoration of intestinal barrier integrity, as evidenced by the upregulation of tight junction proteins and enhanced mucus secretion effectively prevents the leakage of systemic endotoxins [88]. This repair shields the host from M1‐driven inflammatory storms and contributes to systemic immunological equilibrium.

Ultimately, this review suggests that acupuncture may act as a potential eco‐immunological therapy. However, this concept requires further validation. Rather than targeting immune components in isolation, it may harmonize the host's internal ecology. It may do so by restoring the symbiotic balance between the microbiome and the innate immune system. While challenges remain regarding the establishment of direct causality through approaches such as FMT and the systematic quantification of frequency‐specific parameters [35], current research may offer a promising conceptual framework. This framework may help develop personalized, biomarker‐driven protocols. The gut microbiota‐macrophage axis is emerging as a central framework for understanding how peripheral stimulation may translate into sustained host immune homeostasis, though direct causal evidence remains to be fully established.

## Author Contributions

**Tao Yang:** conceptualization, formal analysis, visualization, methodology, project administration, investigation, writing – review and editing, supervision, software, validation, funding acquisition, resources, data curation. **Mei Li:** conceptualization, data curation, formal analysis, visualization, project administration, supervision, investigation, methodology, writing – review and editing, software, validation, resources. **Xiaowen Wu:** conceptualization, data curation, formal analysis, visualization, writing – original draft, project administration, investigation, software, resources.

## Ethics Statement

As a narrative review of previously published literature, this study did not involve direct research on human or animal subjects. Therefore, ethical approval from an institutional review board was not required.

## Conflicts of Interest

The authors declare no conflicts of interest.

## Supporting information


Supporting File

## Data Availability

Data sharing not applicable to this article as no datasets were generated or analysed during the current study.
