# Supplementary material for: Mechanistic Insights Into How Acupuncture Modulates Host Immunity via the Gut Microbiota‐Macrophage Axis
Source: Immun Inflamm Dis. 2026 Nov 5;14(11):e70567. doi: 10.1002/iid3.70567 (PMC13738057; doi:10.1002/iid3.70567)
Supplement: Supplementary file 1 [file IID3-14-e70567-s001.docx]

**Supplementary Table S1. Mapping of mechanistic claims to references, species, study design, and evidence tier.**

**Evidence tier definitions:**

**Tier 1:** Observational rodent studies showing associations.

**Tier 2:** Mechanistic or causal studies, including vagotomy, receptor blockade, depletion, germ-free models, FMT, optogenetics, and electrophysiology.

| Claim ID | Section | Mechanistic claim | Reference(s) | Species | Study design | Evidence tier |
| --- | --- | --- | --- | --- | --- | --- |
| M1 | Introduction | PROKR2-Cre sensory fibers transduce peripheral stimulation into CNS signals that drive the vagal-adrenal axis. | 2 | Mouse | Optogenetic, viral tracing, electrophysiological studies | Tier 2 |
| M2 | Introduction | Acupuncture may operate within the neuro-endocrine-immune network. | 2, 5, 6 | Mouse, Rat | Mechanistic animal studies, including optogenetic, viral tracing, electrophysiological, inflammatory pain, and UC models | Tier 2 |
| M3 | Section 2.1 | Acupuncture may restore alpha diversity and rebalance microbial composition in multiple diseases. | 23, 24 | Rat | Animal models (T2DM rat model; MCAO rat model) | Tier 2 |
| M4 | Section 2.1 | Acupuncture at Back-Shu points may enhance microbial richness and evenness in PCPA-induced insomnia model rats. | 26 | Rat | Metagenomic sequencing in a PCPA-induced insomnia rat model | Tier 2 |
| M5 | Section 2.1 | EA suppresses intestinal ferroptosis and inflammation via Nrf2/HO-1 in obese mice with colitis. | 27 | Mouse | HFD-induced obese mouse model of IBD/colitis | Tier 2 |
| M6 | Section 2.1 | EA at ST36, ST25, and LR3 restores intestinal barrier integrity by modulating mast cell-derived exosomal miRNAs (e.g., miR-149-5p and miR-22-5p) and preventing degradation of tight junction proteins. | 28 | Rat | IBS-D rat model (mechanistic animal study) | Tier 2 |
| M7 | Section 2.1 | EA at BL25 and ST25 suppresses intestinal ferroptosis and inflammation via Nrf2/HO-1 in obese mice with colitis. | 27 | Mouse | HFD-induced obese mouse model of IBD/colitis | Tier 2 |
| M8 | Section 4.1 | EA at ST36 and LI11 increases Bacteroidota and decreases Firmicutes, reducing the Firmicutes-to-Bacteroidetes ratio in diet-induced obese rats. | 29 | Rat | High-fat diet-induced obese rat model (preclinical animal study) | Tier 1 |
| M9 | Section 2.1 | Eight-week EA reduces waist circumference and is associated with changes in the abundance of Akkermansia muciniphila in abdominal obesity. | 30 | Human | Randomized controlled trial | Tier 3 |
| M10 | Section 2.1 | Acupuncture/EA may enrich Lactobacillus and Bifidobacterium and modulate other taxa in rodent models. | 33-35 | Mouse, Rat | Animal models (FC mouse, PSD rat, MCAO rat) | Tier 2 |
| M11 | Section 2.2 | ST36 is densely innervated by sensory fibers, including PROKR2-Cre fibers, which project to NTS and DMV and activate vagal efferents. | 2 | Mouse | Neuroanatomical tracing, optogenetics, electrophysiology | Tier 2 |
| M12 | Section 2.2 | Electroacupuncture attenuates intestinal barrier disruption and inflammation via α7nAChR-mediated HO-1/p38 MAPK/NF-κB signaling in a mouse model of MAFLD. | 38 | Mouse | Mechanistic animal study (mouse model of MAFLD) | Tier 2 |
| M13 | Section 2.2 | Vagal activation may promote epithelial α1,2-fucosylation via Fut2, providing carbohydrate ligands for commensals (non-acupuncture mechanistic evidence). | 39 | Mouse | Mechanistic mouse study (non-acupuncture) | Secondary / Tier 2 (non-acupuncture) |
| M14 | Section 3 | Sodium butyrate activates GPR43 to inhibit NF-κB/MAPK signaling and NLRP3 inflammasome in COPD models. | 43 | Rat and NR8383 cells | In vitro and in vivo mechanistic studies | Tier 2 |
| M15 | Section 3 | LPS/TLR4/NF-κB signaling drives M1 macrophage polarization. | 45 | Mouse | Mechanistic studies (in vitro and in vivo) | Tier 2 |
| M16 | Section 3 | Tryptophan-derived microbial metabolites activate AhR in tumor-associated macrophages and induce an immunosuppressive phenotype. | 46 | Mouse and human | Mechanistic studies | Tier 2 |
| M17 | Section 3 | Activation of the AhR-CYP1A1 axis suppresses M1 polarisation and reduces inflammatory cytokines. | 47 | Mouse cells and animals | Mechanistic studies | Tier 2 |
| M18 | Section 3 | Tryptophan metabolite IAA activates AhR/CYP1A1 and promotes M2 macrophage polarisation. | 48 | Mouse and human cells | Mechanistic studies | Tier 2 |
| M19 | Section 4.1 | Proposed cascade: ST36 → PROKR2 → vagal efferent → butyrate-producing taxa → SCFA → M2 polarization. This is a hypothesis assembled from independent studies; no single study validates the full cascade. | 29, 33, 43, 51 | Rodents (proposed) | Multi-study synthesis (hypothesis) | Hypothesis / Proposed model |
| M20 | Section 4.1 | EA upregulates host-derived miR-205-5p, promotes Lactobacillus reuteri growth, and increases tryptophan metabolites (indole-3-acetamide, indole-3-acetic acid, indole-3-carboxaldehyde) in a mouse model of loperamide-induced constipation. | 33 | Mouse | Loperamide-induced constipation model (mechanistic animal study) | Tier 2 |
| M21 | Section 4.2 | Electroacupuncture restores intestinal barrier integrity, inhibits ferroptosis, and reduces inflammation via Nrf2/HO-1 in HFD-induced obese mice with colitis. | 27 | Mouse | HFD-induced obese mouse model of IBD/colitis | Tier 1 |
| M22 | Section 4.3 | Gut–lung axis: electroacupuncture inhibits NLRP3 inflammasome and remodels gut microbiota in septic mice, alleviating pulmonary and intestinal injury. | 53 | Mouse | Septic mouse model | Tier 2 |
| M23 | Section 5.3 | EA at BL25 and ST25 reduces colonic inflammation and repairs intestinal barrier via Nrf2/HO-1 in HFD-induced obese mice with colitis; no direct M1/M2 polarization data. | 27 | Mouse | HFD-induced obese mouse model of colitis (original animal study) | Tier 1 |
| M24 | Section 5.5 | EA upregulates miR-205-5p, promotes L. reuteri growth, and increases tryptophan metabolites that are known AhR ligands in a mouse model of constipation. | 33 | Mouse | Preclinical animal study | Tier 2 |
| M25 | Section 5.6 | EA at ST36 and ST25 inhibits NLRP3 inflammasome and MAPK pathways, reduces pulmonary and intestinal injury, and remodels gut microbiota in septic mice. | 53 | Mouse | Septic mouse model (original animal study) | Tier 1 |
| M26 | Section 5.2 | The M1/M2 dichotomy is an oversimplification; macrophage states exist along a continuum. | 73 | Human (primary); mouse data integrated | Original research (transcriptome-based network analysis) | Tier 2 |

**Abbreviations:** EA, electroacupuncture; RCT, randomized controlled trial; UC, ulcerative colitis; IBD, inflammatory bowel disease; IBS, irritable bowel syndrome; FMT, fecal microbiota transplantation; WAT, white adipose tissue; AhR, aryl hydrocarbon receptor; HDAC, histone deacetylase; LPS, lipopolysaccharide; NLRP3, NOD-like receptor family pyrin domain containing 3; NF-κB, nuclear factor kappa B.
